# Pattern Analysis of Short Tandem Repeats Allele Frequencies among the Population of Khuzestan Province, South of Iran

**Published:** 2018

**Authors:** Seyed Farzad Hosseini, Mahdi Bijanzadeh, Elham Modheji

**Affiliations:** 1. Department of Forensic Medicine, Khuzestan Legal Medicine Organization, Ahvaz, Iran; 2. Department of Medical Genetics, Faculty of Medicine, Ahvaz Jundishapur University of Medical Sciences, Ahvaz, Iran

**Keywords:** DNA finger printing, Forensic sciences, Genotype, Polymerase chain reaction

## Abstract

**Background:**

The basis of genetic fingerprinting and DNA profiling in forensic laboratories is the use of Short Tandem Repeats (STRs) according to local and ethnical genetics characteristics.

**Methods:**

Forensic parameters and allele frequencies for 15 autosomal STRs in 100 unrelated individuals from Khuzestan province, south Iran were determined. PCR was carried out for amplification of STRs and GeneMapper ID software was used for genotyping and allelic analyzing.

**Results:**

The Power of Exclusion (PE) varied between 0.332 (TPOX) and 0.768 (FGA). With exception of the THO1 (0.020), TPOX (0.014) and D18S51 (0.003), other STRs showed no deviation from the Hardy-Weinberg equilibrium (p>0.05).

**Conclusion:**

Out of 15 STRs, 12 repeats seemed to be more useful and more powerful tools in identity and paternity determination for our studied population. Variation in our data analysis revealed that effective use of these 15 STR loci in forensic cases needed to be localized by collection and analysis of population data from the general population.

## Introduction

The law organizations have greatly benefited from current improvements in the genetic micro and nano-techniques. The advanced forensic laboratories outfitted by bunch of biological evidence from a crime scene to the guilty can reliably exclude falsely accused individuals. In the past three decades, numerous advances in genetic technologies have occurred and most of them included the development of DNA testing and especially Polymerase Chain Reaction (PCR)-based typing methods 1,2.

The basis of genetic fingerprinting and DNA profiling is that twins are the only individuals who have identical copies of the human genome. Of course, the human genome is almost the same in everybody, although it contains many polymorphisms, positions where causes different nucleotide sequence to occur in every member of the population. These polymorphisms include Restriction Fragment Length Polymorphisms (RFLPs), Short Tandem Repeats (STRs), and Single Nucleotide Polymorphisms (SNPs), that can occur within genes as well as in intergenic regions, and altogether there are millions of these polymorphic sites in the human genome. The more powerful technique of DNA profiling is the use of STRs, a short sequence, 1–13 nucleotides in length, which is repeated several times in a tandem array 3. Although for many years the importance of using specific STR for human identification, forensic DNA casework, missing persons, mass disasters, monitoring needle sharing, monitoring transplants, military casualties and so on has been accepted, for detection of their impact for each population, their forensic parameters specially Hardy-Weinberg equilibrium should be calculated 4.

In the population, generally, there might be as many as ten different versions of a particular STR, each of the alleles characterized by a different number of repeats. In DNA profiling, the alleles of a selected number of different STRs are identified. This can be achieved quickly and with very small amounts of DNA by PCRs. The size of the band or bands that are seen in the agarose gel electrophoresis indicate the allele or alleles present in the DNA sample that has been tested. Two alleles of an STR can be present in a single DNA sample because there are two copies of each STR, one on the chromosome inherited from the mother and one on the chromosome from the father 2. Khuzestan province in southwest of Iran has wide ethnical distribution including Fars, Arab, Lur and so on. This study was performed to analyze STR genotypes, report their allele frequencies and evaluate their application among the population of Khuzestan province in Iran.

## Materials and Methods

### Samples and DNA extraction

Blood samples were collected under informed consent, from 100 healthy unrelated men and women (men: 65, women: 35) belonging to three ethnic groups (Arab: 39, Lur: 26, Fars and others: 35) living in Khuzestan province. The research conformed to the provisions of the declaration of Helsinki in 1995 (as revised in Edinburgh 2000) and ethical approval was obtained from the ethics committee of Khuzestan Legal Medicine Organization and informed consent was obtained from all participants. Genomic DNA was extracted by using dried blood samples on FTA cards according to the manufacturer’s instructions 5,6. Laboratory procedures were carried out in genetic laboratory of Khuzestan Legal Medicine Organization.

### PCR and genotyping

For amplification of 15 autosomal STR (CSF1PO, D2S1338, D3S1358, D5S818, D7S820, D8S1179, D13S317, D16S539, D18S51, D19S433, D21S11, FGA, TH01, TPOX, vWA), PCR procedure was performed according to the manufacturer’s instruction (AmpFlSTRIdentifiler™, Applied Biosystems, Foster City, CA, USA), by using the AmpFlSTRIdentifiler PCR Amplification kit. PCR products electrophoresis was performed on an ABI 3500 Genetic Analyser 8-capillary array system (Applied Biosystems, Foster City, CA). 3500 series data collection version 1.0 software was used for data collection. For genotyping and allelic calls, GeneMapper ID software version 3.2 (Applied Biosystems, USA) was used by comparison of samples profile with allelic ladder included in the kit.

### Statistical analysis

Arlequin software version 3.5 was used to calculate allele frequencies and to obtain expected heterozygosity (He), observed heterozygosity (Ho) and probability value (p-value) of Hardy-Weinberg equilibrium 7. PowerStats v1.2 (Promega Corporation) software package was used to obtain statistical parameters of forensic interest including MP, matching probability; PD, power of discrimination; PIC, polymorphic information content; PE, power of exclusion and PI, paternity index 8.

## Results

Forensic parameters and allele frequencies of 15 autosomal STR loci (D8S1179, D21S11, D7S820, CSF-1PO, D3S1358, THO1, D13S17, D16S539, D2S1338, D19S433, vWA, TPOX, D18S51, D5S818 and FGA) are shown in table 1. D19S433 was the most polymorphic autosomal STR, with 15 alleles, while, seven STRs were the least polymorphic with 7 alleles for each (CSF1PO, D3S1358, D13S17, D16S539, vWA, TPOX and D5S818). The most frequent alleles of these two loci had 45.9, 37.2% occurrences for TPOX and CSF1PO, respectively. A PIC value of greater than 0.7 was considered highly informative. Clearly, markers with greater numbers of alleles tend to have higher PIC values and thus were more informative 9. In this study, 13 out of 15 autosomal markers were highly polymorphic with a PIC value of greater than 0.7 (Table 2). Only two markers, D3S1358 and TPOX, with PIC value of 0.64 were shown to be moderately polymorphic. p-value of Hardy-Weinberg equilibrium exact test in three markers including THO1 (0.020), TPOX (0.014), D18S51 (0.003) were lesser than 0.05, so deviation from the Hardy-Weinberg equilibrium was observed (Table 2). The observed heterozygosity percentage (Ho) of 15 STR loci ranged from 11.3 for FGA to 36.7 for TPOX, while it was expected that heterozygosity percentage (He) would vary between 63.3 for TPOX and 88.7 for FGA. Among these 15 STR loci, TPOX with PD= 0.844 and FGA with PD=0.966 had the lowest and highest power of discrimination, respectively. The Power of Exclusion (PE) varied between 0.332 (TPOX) and 0.768 (FGA).

**Table 1.** Allele frequency percentages for 15 autosomal STR loci in 100 samples from Khuzestan province

	**D8S1179**	**D21S11**	**D7S820**	**CSF1PO**	**D3S1358**	**THO1**	**D13S17**	**D16S539**	**D2S1338**	**D19S433**	**vWA**	**TPOX**	**D18S51**	**D5S818**	**FGA**
**5**						1.0									
**6**						34.9						0.5			
**7**			4.1			16.1						1.5			
**8**	1		17.5	0.5		10.9	16.3	2.6	0.5			45.9	0.5	1.6	
**9**	0.5		9.8	1.0		25.0	4.6	17.2		0.5		19.4		8.3	
**9.3**						9.4									
**10**	8.7		22.2	28.1		2.1	3.6	7.8		0.5		5.6	2.0	9.9	
**11**	5.6		25.8	37.2		0.5	30.6	33.9		2.7		24.0	5.1	31.3	
**11.2**										0.5					
**12**	9.2		17.0	28.6	1.0		34.2	22.9		8.2		3.1	12.8	34.9	
**13**	30.6		3.1	3.1			9.2	14.1		20.9			11.7	13.0	
**13.2**										1.6					
**14**	22.4		0.5	1.5	7.7		1.5	1.6		22.5	12.8		19.4	1.0	
**14.2**										4.4					
**15**	15.8				25.0					20.3	13.8		11.7		
**15.2**										4.9					
**16**	3.6				31.1				3.6	6.0	20.4		14.8		
**16.2**										3.8					
**17**	2.0				20.9				21.1	2.2	20.4		6.6		1.6
**17.2**										0.5					
**18**					12.8				9.8		24.0		6.1		1.6
**19**					1.5				16.0		8.2		6.1		5.4
**20**									12.4		0.5		2.0		7.6
**21**									4.1				1.0		17.4
**21.2**															1.5
**22**									3.6						10.9
**23**									13.9						22.3
**23.2**															1.5
**24**									7.7						13.0
**25**									5.2						14.1
**26**									2.1						4.3
**27**		3.6													
**28**		20.1													0.5
**29**		17.0													1.1
**30**		18.0													
**30.2**		5.2													
**31**		3.1													
**31.2**		10.8													
**32.2**		11.3													
**33.2**		9.3													
**34.2**		0.5													

**Table 2.** Forensic parameters for 15 autosomal STR loci in a population sample of Khuzestan province

	**D8S1179**	**D21S11**	**D7S820**	**CSF1PO**	**D3S1358**	**THO1**	**D13S17**	**D16S539**	**D2S1338**	**D19S433**	**vWA**	**TPOX**	**D18S51**	**D5S818**	**FGA**
**MP**	0.083	0.107	0.090	0.090	0.148	0.063	0.043	0.063	0.045	0.108	0.035	0.156	0.067	0.064	0.034
**PD**	0.917	0.893	0.910	0.910	0.852	0.937	0.957	0.937	0.954	0.892	0.965	0.844	0.933	0.936	0.966
**PIC**	0.74	0.71	0.73	0.73	0.64	0.79	0.84	0.79	0.84	0.71	0.87	0.64	0.79	0.79	0.86
**PE**	0.409	0.419	0.350	0.573	0.403	0.569	0.727	0.610	0.510	0.510	0.669	0.332	0.595	0.610	0.768
**PI**	1.6	1.63	1.41	2.33	1.58	2.31	3.73	2.58	2.00	2.00	3.06	1.36	2.47	2.58	4.41
**Ho**	31.3	30.6	35.4	21.4	31.6	21.6	13.4	19.4	25.0	25	16.3	36.7	22.6	19.4	11.3
**He**	68.8	69.4	64.6	78.6	68.4	78.4	86.6	80.6	75.0	75	83.9	63.3	80.6	79.3	88.7
**p-value**	0.448	0.885	0.864	0.984	0.994	0.020	0.405	0.290	0.981	0.789	0.736	0.0141	0.003	0.524	0.067

MP: massively parallel, PD: power of discrimination; PIC: polymorphic information content; PE: power of exclusion; PI: Hardy–Weinberg equilibrium exact test in the study sample: Ho: observed heterozygosity percentage, He: expected heterozygosity percentage.

## Discussion

A set of autosomal STR loci were analyzed in a mixed ethnical population group in Khuzestan province, in order to obtain a genetic characterization of south Iran. Several local studies on molecular genetics loci have been reported in different parts of the world. Hammond *et al* presented a PCR-based DNA-typing method using 13 unlinked STR loci from unrelated individuals presenting to a Houston area blood bank. They reported that this valid statistical analysis is generally simpler than similar analysis of RFLP-VNTR results 10. Two levels of analysis have been devised using two sets of 12 STR loci (D3S1358, vWA, FGA, THO1, TPOX, CSF1PO, D8S1179, D21S11, D18S51, D5S818, D13S317 and D7S820) and 21 STR loci (the former set plus D9S926, D11S2010, D13S767, D14S306, D18S848, D2S1328, D4S243, F13A1, FES/FPS) from north-west African population in 2000. Bosch *et al* analyzed the genetic differentiation among African populations based on their cultural or linguistic differences 11. Hedjazi *et al* in 2013 analyzed allele frequencies for 15 autosomal STR loci in Fars province population, southwest of Iran and by using our similar STR loci, got similar results in Hardy-Weinberg equilibrium, resembling to our heterozygosities. They reported that with exception of D13S317 and TPOX, other STRs were in Hardy-Weinberg equilibrium, same as our report about these two markers in addition to THO1. Fars and Khuzestan are two proximate provinces in Iran, so genetic and ethnic similarities of their population can justify this symmetry 12 and these 12 common STRs can be used for these two ethnic populations. Stanciu *et al* analyzed allele frequencies’ distribution for 15 STRs loci in a population sample of unrelated individuals from the region of Wallachia, South Romania and presented their more genetic similarity 13. Kutanan *et al* in 2014 evaluated the genetic structure and genetic affinity between Thai-Malay Muslims and Thai Buddhists by analyzing 15 autosomal short tandem repeats. They used exact STRs which were used in our study as well 6. Regarding effective results from mentioned researches and current application in forensic laboratories, STRs analysis seem to be a useful and powerful tool in identity and paternity determination.

## Conclusion

Analysis of autosomal STR loci can reveal heterogeneity of geographically and culturally related individuals and different populations, thus, it might be possible to better differentiate populations through the procedure. Today, STRs are used for crime forensic identity, paternity and corpse identity in many related laboratories, while for effective use of these 15 STR loci in forensic cases, it is needed to collect and analyze population data from the general population region in which the crime has happened and adjust them by local characteristics. In order to contribute in construction of STR profiling of the different ethnic groups in geographical areas, similar study with the present population can be useful.
